# Comparative Evaluation of Mathematical Model and In Vivo Study of Calcium Phosphate Bone Grafts

**DOI:** 10.3390/jfb15120368

**Published:** 2024-12-06

**Authors:** Mikhail A. Shlykov, Polina V. Smirnova, Anatoliy S. Senotov, Anastasia Yu. Teterina, Vladislav V. Minaychev, Igor V. Smirnov, Roman A. Novikov, Ekaterina I. Marchenko, Pavel S. Salynkin, Vladimir S. Komlev, Roman S. Fadeev, Irina S. Fadeeva

**Affiliations:** 1 Baikov Institute of Metallurgy and Materials Science, Russian Academy of Sciences, Leninskiy Prospect 49, 119334 Moscow, Russia; ceshakov@gmail.com (M.A.S.); smirnova-imet@mail.ru (P.V.S.); teterina_imet@mail.ru (A.Y.T.); vminaychev@gmail.com (V.V.M.); baldyriz@gmail.com (I.V.S.); 2 Institute of Theoretical and Experimental Biophysics, Russian Academy of Sciences, Pushchino, 142290 Moscow, Russia; a.s.senotov@gmail.com (A.S.S.); salynkin.p.s@gmail.com (P.S.S.); fadeevrs@gmail.com (R.S.F.); 3 N. D. Zelinsky Institute of Organic Chemistry, Russian Academy of Sciences, Leninskiy Prospect 47, 119991 Moscow, Russia; novikovfff@bk.ru; 4 Department of Crystallography and Crystal Chemistry, Faculty of Geology, Moscow State University, 119234 Moscow, Russia; marchenko-ekaterina@bk.ru

**Keywords:** dissolution kinetics, dicalcium phosphate dihydrate, perfusion–diffusion bioreactor, dynamic bilateral material–environment interaction, surface modification

## Abstract

One of the key factors of the interaction ‘osteoplastic material—organism’ is the state of the implant surface. Taking into account the fact that the equilibrium in regeneration conditions is reached only after the reparative histogenesis process is completed, the implant surface is constantly modified. This work is devoted to the numerical description of the dynamic bilateral material–medium interaction under close to physiological conditions, as well as to the assessment of the comparability of the model with *in vitro* and *in vivo* experimental results. The semi-empirical model obtained on the basis of chemical kinetics allows us to describe numerically the processes occurring in the *in vitro* systems and extrapolates well to assess the behavior of dicalcium phosphate dihydrate (DCPD) material under conditions of ectopic (subcutaneous) implantation in Wistar rats. It is shown that an experiment conducted using a perfusion–diffusion bioreactor in a cell culture medium with the addition of fetal bovine serum (FBS) allows for achieving morphologically and chemically identical changes in the surface of the material in comparison with the real organism. This fact opens up wide possibilities for the creation of an analog of a ‘laboratory-on-a-chip’ and the transition from classical *in vivo* models to more controlled and mathematically based *in vitro* systems.

## 1. Introduction

Synthetic calcium phosphate (CaP) materials are commonly used for bone surgery due to their biocompatibility, osteoconductivity and composition close to human bone [1]. These materials offer numerous advantages, including high sterility, a reduced morbidity risk and an inexhaustible supply compared to other bone grafts [2]. However, despite the actively growing market and increasing demand, the clinical effectiveness of CaP materials is significantly inferior to that of grafts and orthobiological materials. The key reason for this is the complexity of the regeneration process, which depends on many factors related to the material and the state of the patient’s organism, as well as the type of bone defect. It is probably impossible to create a universal material: each specific case requires its own unique approach. In order to personalize treatment, a single model of microenvironmental and material interaction is needed for all the many bone grafts (calcium phosphates, bioglass, magnesium and titanium alloys), which will allow scientists to predict the results of a particular approach based on patient-specific data and choose the optimal one.

Many studies, instead of looking for common patterns, aim to compare several materials within a single work. However, for calcium phosphates, for example, more than 10 polymorphic modifications and more than three delivery methods (pastes, cements and granules) are known. This is already 3 × 10 combinations of materials that need to be compared with each other. In addition, biphasic ceramics with different phase ratios can be used. The number of possible material combinations becomes 3 × 10 + 3 × n × ($\frac{10!}{2!\times 8!}$) (let n be the phase ratio variants). Plenty of materials can be doped with ions like Mg^2+^, Sr^2+^, Ba^2+^, Mn^2+^, Zn^2+^, Cu^2+^, Fe^2+^, F^−^, Cl^−^ and CO_3_^2−^ over a wide range of concentrations. Bone grafts can differ in morphology—micro- and macrostructure—which significantly affects the cellular response. Comparisons can be conducted *in vitro* on different cell lines, e.g., MC3T3-E1, MG-63 and hBMSCs, and *in vivo* on different animals: rats, horses, dogs, pigs and monkeys. Thus, it can be seen that it is extremely difficult to find the optimal calcium phosphate bone grafts using empirical methods—a unified model of calcium phosphate behavior under physiological conditions should be developed, which for each material will differ only in constants.

The behavior of implants in liquid media has been widely studied by the scientific groups of Nancollas G.H. et al. [3] and Christoffersen J and Christoffersen M.R. et al. [4]. In their studies, the authors reported kinetic equations, measured and calculated constants for many phosphates and created a theoretical basis for further work. However, the authors did not extrapolate their research to real systems, such as a culture medium or blood. Research into the resorption behavior of CaP materials has continued and the rates of precipitation and dissolution of CaP as a function of polysaccharide and organic acid concentrations have been studied [5,6]. The deposition rates of CaP phases in various compositions of Simulated Body Fluid (SBF) were studied [7,8,9]. The interaction between Ca^2+^ and tris(hydroxymethyl)aminomethane (Tris) in SBF solutions was analyzed [8]. In 2019, data from the group of Rabajieva D. et al. were published, in which the phase transformation of dicalcium phosphate dihydrate (DCPD) to octacalcium phosphate (OCP) in an SBF solution was modeled, but without numerical data—there were no constants or rates [10]. Despite the large number of works, we could not find any studies devoted definitively to the numerical description of physiological processes.

In addition to the dissolution of CaP, after the implant enters the biological environment, the material sorbs proteins and amino acids, which constitute 7–8 wt.% of blood plasma [11]. A number of studies have shown that fibrins, fibronectins, bone morphogenetic proteins and some synthetic peptides can regulate cell adhesion and subsequent tissue attachment to materials used as implants—this promotes normal tissue regeneration [12,13]. Proteins are known to undergo conformational changes upon adsorption and most material–cell interactions will depend on the nature of these conformational modifications [14]. Therefore, the evaluation of material–protein interactions should be given special attention.

Human (or bovine) serum albumin (HSA (BSA), 0.4–0.8 mM, approximately 50% of the total human blood protein concentration) is an excellent model protein for studying such interactions. Albumin plays an important role in the distribution of calcium ions in the body [11,15]. At physiological calcium concentrations, only three selective Ca^2+^ binding sites are active in albumin, all of which are located in domain I [16]. This circumstance is important to take into account when modeling the dissolution of calcium phosphate materials in the extracellular environment of the organism and the effect of the calcium concentration on the cellular response as well as the biological properties of the newly formed surface.

The mechanism of protein binding to the surface of the material can be described either by computer simulation—the molecular dynamics method [17]—or in terms of chemical kinetics [18]. Due to adsorption in the etching wells on the surface of CaP, proteins can reduce the rate of calcium ion release into solution, thus slowing down the dissolution of materials [18,19], as well as lowering the rate of crystallization, increasing the crystallite size and reducing the crystallinity of the new phase precipitated from the solution [20,21]. What is important is the fact that the chemistry of the process does not change: no new phases are formed [22]. Apparently, due to the inhibitory effect of proteins, fetal bovine serum (FBS), a model system widely used in cell engineering, also stabilizes high CaP supersaturation values (preventing spontaneous mineralization) [23,24,25].

It can be concluded that many elementary parts of the regeneration process have been carefully studied, described and modeled for a wide range of compositions and conditions. However, a single mathematical model summarizing all solution–medium interactions has not yet been proposed.

In addition, it is crucially important to take into account the cell factor. And, to do so, researchers should use the proper experimental techniques. In recent years, many attempts have been made to develop experimental systems that reflect bone metabolism and the role of the vascular network. Obviously, these processes cannot be displayed in conventional 2D monocultures (in plates and Petri dishes). In particular, in conventional 2D cultures it is impossible to reflect cell—organic (mainly collagen)—and inorganic (mainly hydroxyapatite) substrate interactions on the culture plate, while it is the bone matrix that regulates the functions and differentiation of bone cells [26,27]. Modeling a bone defect *in vitro* requires the consideration of the paracrine and mediator interactions between vessel-forming endothelial cells, blood cells, bone-forming cells and bone-resorbing cells. In the case of a fracture, additional factors become important, such as the migration of immune cells, which represent the main drivers of successful bone regeneration [28,29]. Co-culture models differ not only in the type of cells used. There are also significant differences in individual co-culture setups:

- Not all co-cultures reflect direct intercellular interaction [30];
- Smaller amounts provide cell–matrix interaction [30,31,32];
- Few models preserve the natural 3D organization of cells [32,33];
- Single works account for the mechanical stimulation of bone cells [34].

Blood or tissue fluid flow can be modeled (and, consequently, the volume of the medium and the concentrations of dissolved substances can be kept constant; perfusion and mechanical impacts on cells can be simulated) using perfusion–diffusion-type bioreactors, which ensure the removal of restrictions on mass exchange and mass transfer directly in the zone of contact between the material and the liquid medium [35]. This is necessary for both nutrient supply and waste disposal to maintain cell viability in large three-dimensional (3D) assemblies, which experimental systems for the evaluation of bioactive materials appear to be [36].

The logical approach for approximating the model to the real conditions of the organism is the use of a mixture of 10% blood serum (in our case, fetal (embryonic) bovine serum, FBS) and 90% culture medium, DMEM (Dulbecco Modified Eagle’s Medium). This ratio is widely used in cell investigations: DMEM provides the necessary amino acids and nutrients for cell development, while embryonic serum provides the necessary growth factors and proteins for proliferation (without hormones and immune cells) and protects cells from turbulent shear stresses realized in the bioreactor [37]. This solution composition will allow us to study the influence of organic components on the behavior of the material, as well as to include the cellular factor in the experiment without changing the solution composition.

A full understanding of the reactions that occur (and, accordingly, full control over the direction of the processes) is possible only with mathematical modeling. Thus, this work aims to create a numerical description of the CaP behavior at every stage of simulation: from simple static inorganic systems to dynamic systems with a composition close to human blood (in the future, cellular systems and actual organisms). This model is the first step to understanding and quantitatively assessing the processes of the biointegration of materials, and the first link in the stage-by-stage modeling (mathematical and experimental) of a bone defect *in vitro*, without the use of animals.

Dicalcium phosphate dihydrate CaHPO_4_∙2H_2_O (DCPD, brushite) was used in this study as a well-described CaP with a relatively high resorption rate and a good immune response *in vivo* [38,39].

## 2. Materials and Methods

### 2.1. Synthesis Procedure and Characterization

#### 2.1.1. Material Synthesis

All reagents were purchased from Sigma-Aldrich (St. Louis, MO, USA) unless otherwise stated. The synthesis of low-temperature DCPD has been developed in detail and is well described in the literature [40]. α-TCP powder was initially prepared by mixing a 0.5 M solution of (NH_4_)_2_HPO_4_ and a 0.5 M solution of Ca(NO_3_)_2_∙4H_2_O in a Ca/P = 1.67 ratio according to the reaction

3Ca(NO_3_)_2_∙4H_2_O + 2(NH_4_)_2_HPO_4_ + 2NH_4_OH → Ca_3_(PO_4_)_2_ + 6NH_4_NO_3_ + 6H_2_O (1)

**Figure 1:**
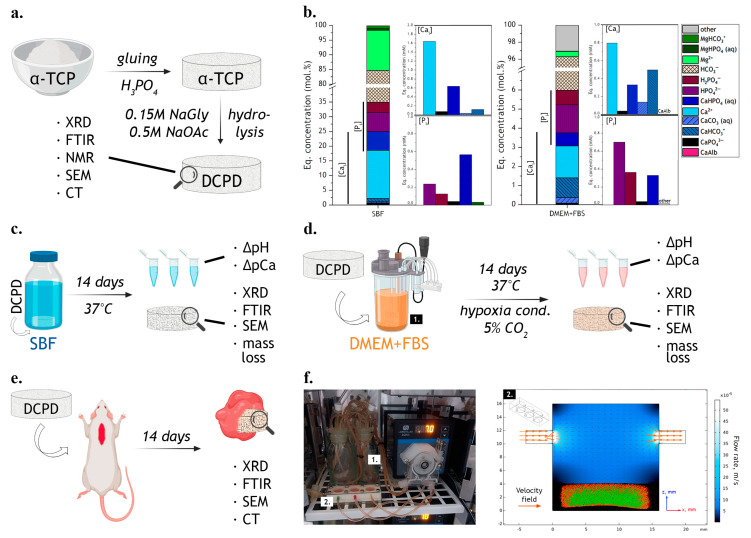
Scheme of the experiment: (**a**) the method of synthesis; (**b**) the characteristics of the media used: the distribution of ionic forms in SBF and in 90% DMEM + 10% FBS; (**c**) the scheme of *Model I* and the methods of analysis used; (**d**) the scheme of *Model II* and the methods of analysis used; (**e**) the scheme of the *in vivo* experiment and the methods of analysis used; (**f**) the appearance of a bioreactor and the velocity distribution in a culture cell.

The value of pH = 7.00 ± 0.05 was maintained throughout the synthesis by the addition of NH_4_OH. α-TCP powder was thermostatically dried at 60 °C and glued into tablets by chemical bonding with a few drops of H_3_PO_4_ (RusChem, Saint-Petersburg, Russia). DCPD tablets were obtained by soaking α-TCP tablets in a solution containing 1.5 M sodium acetate and 0.15 M L-glutamic acid (pH = 5.5 ± 0.05), with a mass ratio of sample/solution = 1/100, for 24 h at 37 ± 1 °C and with constant stirring (Figure 1).

#### 2.1.2. Phase Composition

The phase composition of the samples was studied through X-ray powder diffraction on an XRD-6000 diffractometer (Shimadzu, Kyoto, Japan) using Cu Kα radiation, through infrared spectroscopy and through solid-state NMR spectroscopy. The internal standard method was used to assess the presence of the amorphous phase: XRD was performed on a 70%/30% mixture of the sample/α-Al_2_O_3_ (corundum), then the RIR method was used to compare theoretical and known ratios of the sample/α-Al_2_O_3_. IR spectra were obtained through attenuated total reflection Fourier IR spectroscopy (FTIR-ATR) on a Vertex 70v vacuum spectrometer (Bruker, Billerica, MA, USA) in the range of 400–4000 cm^−1^ with a resolution of 4 cm^−1^ on the grazing platform angle specular reflectance. Solid-phase NMR spectra were recorded on a AVANCE III WB 400 MHz spectrometer (Bruker, Billerica, MA, USA). To confirm the crystal structure and the monophasic nature of the synthesized materials, Rietveld calculations using Jana2006 (v. 20/02/2023, Institute of Physics, Prague, Czech Republic) and density functional theory calculations (DFT calculations) using Quantum Expresso 5.2.1 software were performed.

#### 2.1.3. Microstructure and Morphology

The surface morphology was investigated using SBU II SEM (Tescan Vega, Brno, Czech Republic). The SKYSCAN 1275 micro-CT scanner (Bruker, Billerica, MA, USA) with 4.5 μm expansion was used to analyze morphological and density characteristics of the materials using Bruker-MicroCT CT-Analyser (“CTAn”) (v. 1.19.11.1, Bruker, Billerica, MA, USA). The surface area was measured using the BET nitrogen adsorption method on a Tristar II analyzer (Micromeritics, Norcross, GA, USA).

### 2.2. Solubility Experiment

#### 2.2.1. Model I

*Model I* is a solubility experiment in a static non-organic system according to ISO 10993-1-2011 [41] on the degradation assessment of biomedical products. The dissolution kinetics were evaluated in an isotonic buffer solution SBF, with a mass ratio of sample/solution = 1/100. SBF simulates the inorganic component of human blood serum and was used as the first point in a step-by-step approximation of the experimental model to the real organism conditions. SBF was prepared according to Table 1. The pH was maintained using a Tris-HCl (RusChem, Saint-Petersburg, Russia) buffer system.

PET containers with solutions and samples were kept in the thermostat at 37.0 ± 0.1 °C for up to 14 days. Daily potentiometric measurements were carried out using a pH meter (Econix-expert, Moscow, Russia) with a combined electrode ESC-10601 (Anion, Novosibirsk, Russia) and ionmeter Anion-4130 (Anion, Novosibirsk, Russia) with a calcium-selective electrode ELIS-121 (Anion, Novosibirsk, Russia). Changes in the phase composition and morphology were evaluated using XRD analysis, IR spectroscopy, SEM and CT as described above.

#### 2.2.2. Model II

*Model II* was a solubility experiment in a dynamic organic system using a mixture of 90% DMEM culture medium (PanEco, Moscow, Russia) + 10% fetal bovine serum (FBS, PanEco, Moscow, Russia). This solution composition allowed us to study the effect of organic components on the behavior of the material, as well as to include the cellular factor in the experiment without changing the solution composition. Every day, one-half of the solution was retrieved for sampling. The experiment was conducted at t = 37.0 ± 0.1 °C and pH = 7.4 ± 0.1 in a bioreactor in a CO_2_ incubator, under hypoxia conditions (5% CO_2_ in the air) and with a sample/solution ratio = 1/100.

In the present work, a perfusion–diffusion bioreactor was used to simulate the blood and interstitial fluid flow at the site of material implantation, imitating the dissolution and related phase transformations of calcium phosphate materials and reproducing the *in vitro* conditions of the organism (currently without cells) as close as possible. This bioreactor is a compact closed system consisting of a conductive system, gas bubble traps, media tanks (Figure 1(f1)), culture cells (Figure 1(f2)), and a peristaltic pump. The compactness of the system allows the bioreactor to be placed in a laboratory CO_2_ incubator, providing humidity control and a gas phase composition close to *in vivo* conditions (5% CO_2_). The optimal flow rate for the bioreactor used in our work was 600 µL/min, which logically fits into the recommended range of optimal values of the flow rate, which according to the literature is from 200 to 1000 µL/min [43].

### 2.3. Cell Culture

Mouse embryonic fibroblast cells, C3H/10T1/2, were obtained from the ATCC (Manassas, VA, USA). These cells were grown in the EMEM growth medium (Sigma-Aldrich, Milwaukee, WI, USA), enhanced with heat-treated FBS (Gibco, Waltham, MA, USA) up to a final concentration of 10% and 40 μg/mL gentamicin sulfate (Sigma-Aldrich, St. Louis, MO, USA), under conditions of 5% CO_2_ content in the atmosphere and at 37 °C. The cell cultures were screened for mycoplasma contamination utilizing the MycoFluor™ Mycoplasma Detection Kit, and no evidence of mycoplasma was found.

### 2.4. Cytotoxicity Assays

For *in vitro* studies, mouse embryonic fibroblast cells C3H/10T1/2 were placed in amounts of 5 × 10^3^ cells in 100 microliters of full growth medium into 96-well plates. Following 24 h of cultivation, the medium was replaced with 100 microliters of a medium containing the aforementioned DCPD at concentrations of 10, 3, 1 and 0.3 mg/mL, and the cultivation proceeded for 24 and 72 h. Control conditions involved growing the cells in media without added DCPD. Next, the cell viability was assessed along with morphological analyses of the cell culture environment.

The cytotoxicity of DCPD and the morphological condition of the cell culture were evaluated through the *in vivo* labeling of cells with fluorescent dyes such as Hoechst 33,342 (highlights the blue nuclei of both living and dead cells), propidium iodide (highlights the red nuclei of dead cells), and calcein AM (highlights the green cytoplasm of living cells). Cells were labeled by incorporating 1 μg/mL Hoechst 33,342 (Sigma-Aldrich, St. Louis, MO, USA), 1 μg/mL propidium iodide (Sigma-Aldrich, St. Louis, MO, USA) and 2 mM calcein AM (Sigma-Aldrich, St. Louis, MO, USA) into the culture medium. Labeling occurred inside a CO_2_ incubator for 30 min at 37 °C and with 5% CO_2_ content in the air.

The microscopic analysis of the dyed cell cultures and photomicrographs was carried out using a Nikon Eclipse Ti-E (Nikon, Tokyo, Japan). The plate containing the cells and test specimens was then moved to a microscope chamber maintained at 37 °C and a 5% CO_2_ concentration. Cytotoxicity assessment entailed determining the number of live and dead cells per visual field using the ImageJ software (v. 1.53k, University of Wisconsin, Madison, WI, USA).

### 2.5. Implantation of Materials in Laboratory Animals

Sexually mature males of Wistar stock, weighing 200 ± 10 g, were used in the experiment. The study of the phase transformations of materials under *in vivo* conditions was carried out through the ectopic (subcutaneous) implantation of samples in rats in the area of the midline of the back according to ISO 10993-6-2023 [44] using the following procedure. Before surgery, animals were anesthetized through the intramuscular injection of Zoletil (6.0 mg/1 kg body weight) and xylazine (10.0 mg/1 kg body weight) into the outer thigh muscles (each drug into a different muscle). The anesthetic depth was assessed by pinching the middle finger of the rats’ hind limb with tweezers; the surgical procedure was only started if there was no pedal withdrawal reflex. Then, the back area between the shoulder blades was cleaned of hair and the skin was treated with an antiseptic solution (70% ethyl alcohol). Then, under the conditions of the operating box, the skin was lifted over the back muscles with forceps (pinching motion) and dissected with a transverse incision (1 cm long) using straight blunt-pointed scissors. Curved vascular blunt-tipped scissors were applied to the skin incision towards the central back (with a slight deviation towards the left edge) and the subcutaneous space was bluntly pushed parallel to the skin to form a subcutaneous pocket into which the DCPD tablet was placed and spread by palpation. The skin incision was sutured with lavsan (4/0 USP) using simple surgical sutures and treated with an antiseptic solution, and a sterile self-adhesive dressing was applied.

In the postoperative period, the animals were kept under standard conditions. Animals were grouped into four individuals in Type 4 cages (1820 cm^2^). The polycarbonate cages were equipped with steel louvered lids with a feed recess, steel feed dividers and steel label holders. The cages were additionally provided with environmental enrichment material—red transparent polycarbonate houses (Tecniplast S.P.A., Buguggiate, Italy). Throughout the implantation period, the animals were continuously monitored clinically with the recording of possible adverse signs and body weight control.

Animals were removed from the experiment in accordance with the requirements of ISO 10993-2-2022 [45] using humane euthanasia (CO_2_ protocol) after 7 and 14 days of implantation, followed by the careful extraction of the material. To prevent possible phase transformations, DCPD tablets were cleaned from tissues, washed 3 times with distilled water, dried in air at 37 °C and analyzed according to Figure 1e.

### 2.6. Mathematical Modeling and Statistical Analysis

Regression analysis was performed using Matlab 2022b software (v. 9.13.0.2049777, The MathWorks Inc., Torrance, CA, USA). The velocity distribution in the culture cell of the bioreactor (Figure 1f) was calculated using Comsol Multiphysics software (v. 6.1, COMSOL Inc., Burlington, MA, USA).

Results *in vitro* are presented as the mean ± standard deviation (M ± SD). Each *in vitro* and *in vivo* experiment was performed at least five times (*n* ≥ 5). The collected findings were statistically analyzed using the Python 3 programming language (ver. 3.10.6) in the Spyder framework for development (ver. 5.4.1), with the Pandas (ver. 1.5.3), Numpy (ver. 1.23.5) and Scipy (ver. 1.10.0) tools.

## 3. Results

### 3.1. The Physico-Chemical Characteristics of Low-Temperature DCPD

**Figure 2:**
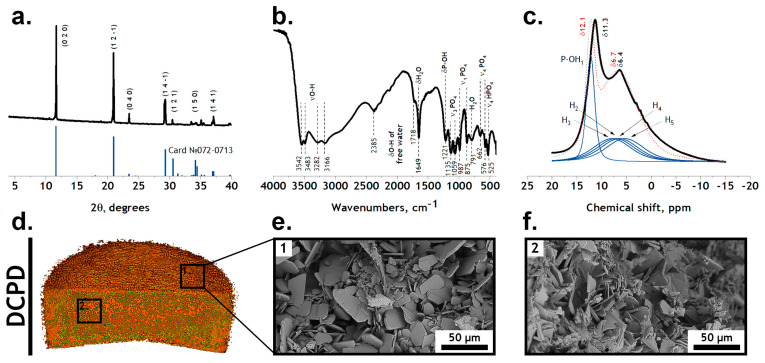
Crystal structure and morphology of dicalcium phosphate dihydrate (DCPD) samples: (**a**) XRD, (**b**) FTIR and (**c**) solid-state NMR (black—experimental spectrum, blue—calculated components, red—cumulative spectrum) spectra; (**d**) micro-CT and (**e**,**f**) SEM images.

According to the XRD, FTIR and solid-state NMR results (Figure 2), a single-phase high-crystalline DCPD was obtained. The IR spectrum of DCPD shows bending modes of the PO_4_^2−^ group at 875 and 987 cm^−1^ (ν1); 1059 and 1135 cm^−1^ (ν3); and 525, 576 and 662 cm^−1^ (ν4). Peaks assigned to the P-OH bound were noted at 875 and 1212 cm^−1^. The stretching modes of the O-H bond of lattice water appear at 3550–3160 cm^−1^, with bending modes at 1649 cm^−1^. The presence of absorbed water was observed at 2385 and 1718 cm^−1^ [22]. The XRD spectrum corresponds to card № 72-0713 of the XRD base ICDD (Powder Diffraction File, Alphabetical Index Inorganic Compounds, Pennsylvania: JCPDS, 1997). The main XRD peaks of DCPD ((020), (02-1), (040) and (14-1)) were noted. Full-profile analysis was performed using Jana2006 software and CIF files from the Crystallography Open Database (COD ID 1533075). The resulting parameters are shown in Table 2.

Brushite crystallizes in the Cc space group. The unit cell of DCPD contains five protons: four of them belong to two water molecules; one is bound to oxygen in the hydrophosphate group. The most intense peak in the ^1^H MAS NMR spectrum is at 11.3 ppm and belongs to the P-OH group. The peak at 6.4 ppm is a superposition of four overlapping peaks of nonequivalent water molecules and is poorly resolved in the current imaging geometry.

Similarly to the XRD spectrum, it is possible to obtain a theoretical NMR spectrum from the data of the crystal structure of a substance. The structure after full-profile analysis was also optimized using DFT calculations in Quantum Expresso (QE) with the following parameters: $E_{cutoff}=80\;Ry,\;k_{Br}=4\times 4\times 4,$ and PBE-kjpaw pseudopotentials. Then, using the GIPAW approach from QE, the chemical shift tensor for the DCPD structure was calculated according to [46]. The resulting spectra are highlighted in blue in Figure 2c. The discrepancy between the calculated and experimental spectrum may be due to low values of $E_{cutoff}\;\mathrm{and}\;k_{Br}$, as well as different calibrations, temperature factors and adsorbed water or pseudopotentials that were too soft. Nevertheless, very good agreement between the calculated and measured XRD and NMR spectra proves the monophasic and high-crystalline nature of our material, which is crucial for the following experiments and calculations.

The microstructure of the obtained samples is typical for DCPD: plate-like crystals with an average size of 19.5 ± 2.1 µm.

### 3.2. Interaction of DCPD with Non-Organic Solutions

**Figure 3:**
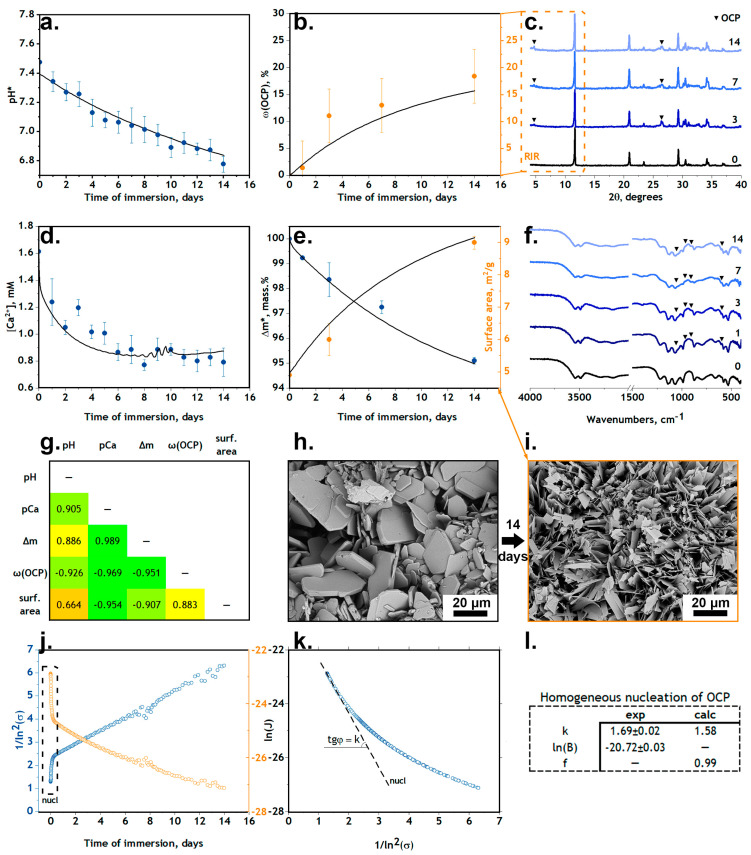
Interaction of DCPD with non-organic solutions: scatters—experimental data; lines—calculations from *Model I*. (**a**) pH change value in SBF solution; (**b**) change in mass ratio of OCP in samples; (**c**) XRD results: 0-3-7-14 days of immersion; (**d**) concentration of free calcium ions in solution; (**e**) change in mass and surface area of samples; (**f**) FTIR spectra; (**g**) correlation matrix of measured parameters; (**h**,**i**) SEM images of sample surface microstructure before and after degradation; (**j**,**k**) region of homogeneous nucleation; (**l**) comparison of calculated values and approximation results. Asterisks * denote the primary parameters for optimization; the rest of the data were used to verify the optimized parameters (see text).

SBF was chosen in this work as an inorganic solution simulating the extracellular fluid of the organism. The phase transformation DCPD→OCP was observed for DCPD samples in the SBF solution, which is in agreement with the CaP stability diagram [3]. Although hydroxyapatite precipitation is thermodynamically more favorable, such a process is kinetically difficult—OCP precipitation is much faster [47]. Meanwhile, the introduction of various impurities, such as fluoride ions, into the system can shift the equilibrium towards the formation of apatite [48]. The presence of the second phase is clearly evidenced by the XRD results: on the third day, a peak at θ = 4.72°, characteristic for the (010) OCP plane, could be detected. The mass fraction of the formed OCP can be calculated from the ratio of the intensities of the peak (010) of OCP and the peak (020) of DCPD using the corundum number method (RIR method, Figure 3b,c) [49]:

(2) ωOCP=I010RIROCPI020RIRDCPD +I010RIROCP, RIRDCPD=1.42,RIROCP=0.5

The fact of phase transformation agrees with the results of IR spectroscopy. The modes characteristic of OCP became noticeable on the third day: vibrations at 561, 602, 962 and 1040 cm^−1^ belong to PO_4_^3−^ ions, vibrations at 916 cm^−1^ and 1121 cm^−1^ to HPO_4_^2−^ groups. The absence of an amorphous phase was confirmed through XRD analysis using the internal standard method, which involves mixing the sample and α-Al_2_O_3_ in a given mass.% and comparing the intensities of theoretical and experimental spectral lines [49].

The modification of the surface morphology is clearly visible in SEM images (Figure 3h,i). At the initial time point, the surface structure displays the characteristic DCPD plates with an average size of 19.5 ± 2.1 μm. After soaking the samples in model liquids, the dissolution of large DCPD plates and the growth of OCP crystals with an average size of 4.8 ± 0.6 μm on their surface were observed. Newly formed crystals had the characteristic OCP shape of thin sharp plates, which can potentially have a cytotoxic effect due to the possibility of damaging the cell membrane [50]. According to BET nitrogen adsorption measurements, there was a doubling of the surface area of the material (Figure 3e).

Due to recrystallization (Equation (3)), there was a decrease in the samples’ weight, as well as a decrease in the pH value of buffer solutions (Figure 3a,e):

(3) $$\{\begin{array}{c} CaHPO_{4}\cdot 2H_{2}O\;\to \;Ca^{2+}+HPO_{4}^{2-}+2H_{2}O\;\\ 8Ca^{2+}+6HPO_{4}^{2-}+5H_{2}O\to Ca_{8}{\left(HPO_{4}\right)}_{2}{\left(PO_{4}\right)}_{4}\cdot 5H_{2}O↓+4H^{+} \end{array}$$

The obtained experimental data can be interpreted based on the postulates of chemical kinetics and the studies [3,4,7,51,52,53,54]. The development of the model consisted of several points:

(1) The calculation of the distribution of ionic forms in the SBF solution according to Table 1 and Equation (4).
(2) The determination of ion activities.
(3) The calculation of the thermodynamic driving force.
(4) The application of a semi-empirical model to describe the kinetics of the processes.
(5) The comparison of *Model I* and the data obtained for a static inorganic system.
(6) The applicability of *Model I* to the description of a dynamic inorganic system.
(7) The comparison of the semi-empirical model from (4) and the classical theory of nucleation.
(8) The applicability of *Model II* to the description of a dynamic organic system.

To determine the equilibrium concentration of an individual ion unit, all possible association/dissociation reactions in the SBF were taken into account. In total, 15 association/dissociation reactions and four mass balance equations were used to calculate the equilibrium concentrations of all of the ion units in the SBF (for $Ca^{2+}$, $Mg^{2+}$,$\;P_{i}\;\mathrm{and}$ ${{CO}_{3}}^{2-}$). The association/dissociation reactions are listed in Table 1. Mass balance equations, for example, for $Ca^{2+}$, are given as follows:

(4) $$C_{Ca^{2+}}=\left[Ca^{2+}\right]+\left[CaPO_{4}^{-}\right]+{\left[CaHPO_{4}\right]}_{\left(aq\right)}+\left[CaH_{2}PO_{4}^{+}\right]+\left[CaHCO_{3}^{+}\right]+{\left[CaCO_{3}\right]}_{\left(aq\right)}+\left[CaOH^{+}\right],$$

where $C_{Ca^{2+}}$ is the theoretical concentration from Table 1 and [$Ca^{2+}$] is the equilibrium concentration of the respective form [7]. The solution is graphically represented in Figure 1b.

It should be noted that for the system H_3_PO_4_—PO_4_^3−^ in this work, in contrast to the works [52,53,54], equilibrium constants were used for ionic strengths of 0.15–0.2 M and a temperature of T=37 °C (Table 1), calculated according to the equation $pK^{\prime }=pK{}^{\circ}-a\sqrt{I}+bI$ [42]; a=1.56 and  b=1.22.

For an electrolyte solution, ion interactions reduce the effective concentration of free ions. This phenomenon is described in terms of activity: $a_{i}=\gamma_{i}{\left[C\right]}_{i}$, where $\gamma_{i}$ is the activity coefficient and ${\left[C\right]}_{i}$ is the equilibrium concentration of the i-th ion. The activity coefficient can be calculated from the Debye–Hückel equation refined by Davis [7,42,52,53,54]:

(5) $$lg\gamma_{i}=-A\times z_{i}\times \left(\frac{I^{\frac{1}{2}}}{1+I^{\frac{1}{2}}}-0.3I\right)$$

In Equation (5), $\gamma_{i}$ is the activity coefficient of an ion with a charge of z_i_, I is the ionic strength of solutions (Equation (5) valid in the range of 0.1M≲I≲0.3M) and A is the temperature-dependent constant, taken as 0.5231 at 37 °C [7]. The ionic strength can be calculated by the classical equation (Table 1)

(6) $$I=0.5\times \sum C_{i}z_{i}^{2}$$

The processes occurring in the system can be characterized as a combination of dissolution–crystallization processes [3]. The thermodynamic driven force in this case is given as follows:

(7) $$\frac{\Delta \mu }{kT}=\frac{1}{n}ln\frac{a_{i}}{a_{i}^{e}}=\frac{1}{n}ln\frac{{\left(a_{A^{x+}}\right)}^{y}{\left(a_{B^{y-}}\right)}^{x}}{K_{sp}}=ln\left(S\right)$$

where k is the Boltzmann constant, n is the number of ion units in a CaP molecule, $a_{i}^{e}$ is the activity of the *i*-ion at the equilibrium and S is supersaturation that is defined by the ratio of the activity product of ion units composing precipitates to the corresponding solubility product ($K_{sp}$) [3]. In the SBF solution, $S_{DCPD}<1$ (Table 1), so DCPD is dissolving. $S_{OCP}>1$; therefore, OCP precipitation occurs.

Chemical reactions in our case are described by the approximate system Equation (3). A commonly used reciprocal of supersaturation to undersaturation is represented as follows:

(8) $$\{\begin{array}{c} \sigma_{\mathrm{DCPD}}=1-S=1-{\left(\frac{a\left(Ca^{2+}\right)a\left(HPO_{4}^{2-}\right)}{K_{sp}}\right)}^{\frac{1}{2}}\;\\ \sigma_{\mathrm{OCP}}=1-{\left(\frac{a{\left(Ca^{2+}\right)}^{8}a{\left(HPO_{4}^{2-}\right)}^{2}a{\left(PO_{4}^{2-}\right)}^{4}}{K_{sp}}\right)}^{\frac{1}{14}} \end{array}$$

The rates of chemical processes can be described by a semi-empirical system:

(9) $$\{\begin{array}{c} \frac{d\left[\mathrm{DCPD}\right]}{dt}=J_{\mathrm{DCPD}}=k_{1}s_{1}{\left(\sigma_{\mathrm{DCPD}}\right)}^{n1}\\ \frac{d\left[\mathrm{OCP}\right]}{dt}=J_{\mathrm{OCP}}=k_{2}s_{2}{\left(\sigma_{\mathrm{OCP}}\right)}^{n2} \end{array}$$

where k_i_ is the kinetic coefficient, s_i_ is the specific surface area, K_sp_ is the solubility product of DCPD and OCP ($2.2\times 10^{-7}\;\mathrm{and}\;2.5\times 10^{-99}$ at 37 °C, respectively [51,53]), n_i_ is the effective reaction order and $\frac{d\left[\mathrm{CaP}\right]}{dt}$ is the time dependence of the amount of dissolved/crystallized CaP.

Two approximations were made for further modeling. Firstly, based on SEM images (Figure 3h,i), it can be assumed that the surface of DCPD is uniformly covered by a layer of OCP crystals. Accordingly, the surface area s_1_ in Equation (9) is a function of several variables $s_{1}=s_{1}$($J_{\mathrm{DCPD}},\;J_{\mathrm{OCP}},\;t)$. We suppose that the DCPD surface area (DCPD–solution contact area) decreases according to a linear law (Equation (10)). There are no crystallization inhibitors in the system (such as glutamic acid in OCP synthesis); therefore, many small OCP crystals are formed (as seen in the SEM images, Figure 3h,i) and the total surface area of the whole sample increases. This process is also described by a linear relationship:

(10) $$\{\begin{array}{c} s_{1}=s_{1}{}^{\circ}-m_{OCP}\times \delta \\ s_{2}=s_{2}{}^{\circ}+m_{OCP}\times \delta \end{array}$$

where $s_{i}{}^{\circ}$, the initial surface area of DCPD and OCP, correspondently, $m_{\mathrm{OCP}}$, the mass of crystallized OCP, and δ, some proportionality coefficient, were some of the optimized parameters in the model. A linear dependence was also proposed by the authors of [55] and it successfully described the experimental data. 

The second approximation was the assumption that the rate constant and effective orders of reaction are independent of the pH of the system, $k_{i}\neq k_{i}\left(pH\right)=const\;\mathrm{and}\;n_{i}\neq n_{i}\left(pH\right)=const,$ since there are strong protein and carbonate buffer systems present in the real organism that prevent a significant change in the pH value.

The model was optimized using the least squares method and simulated annealing algorithm using the experimental values marked with an asterisk * in Figure 3. The results for the mass change of the samples were used as the basis for the model, as the gravimetric method is a standardless method and therefore the most accurate of the available analytical methods. Optimization was carried out according to three parameters: k_i_ and δ; the minimized function was $\varphi =\underset{k}{\sum }W_{k}{\left(J_{k}^{exp}-J_{k}^{calc}\right)}^{2}$, where $W_{k}$ is the standard deviation of the given experimental value $J_{k}^{exp}$. Taking into account the physical meaning of the effective reaction orders, n_i_, they were equated to integers: $n_{DCPD}=1$ and $n_{OCP}=4$ [52,53]. There are several possible mechanisms: n = 1–2 for combined mechanisms—adsorption + surface diffusion, bulk diffusion + polynuclear mechanism, etc.; n = 2 for surface diffusion and integration; and n > 2 corresponds to polynuclear growth + spiral growth [56,57,58]. 

According to the optimization results, the following values were obtained: $pk_{DCPD}=6.87\frac{g}{s\times m^{2}}$, $pk_{OCP}=7.04\frac{g}{s\times m^{2}}$ and $\delta =25.01\frac{m^{2}}{g}$. The remaining data (pCa, ω(OCP), surface area) were used to verify the obtained results.

Figure 2e confirms the validity of our assumptions: the values of the material surface area calculated by Equation (10) correlate well with the experimental data obtained in the SBF solution.

Thus, *Model I*, using constants of material parameters (s_i_, K_sp_) and solution parameters (C_i_, pH_0_, T), is able to describe the main aspects of the bilateral material–medium interaction at the early stages (up for 14 days) of degradation. 

The obtained semi-empirical system, Equation (9), can also be interpreted within the classical theory of crystallization. In order for crystallization to begin, a critical nucleus with a radius of $r_{c}=\frac{2V_{m}\gamma_{OCP}}{RTln\left(S\right)}$ should form, i.e., the energy gain from the formation of new bonds should be greater than the energy loss for the formation of a new surface. The nucleation rate is described by the equation [59]

(11) $$J_{OCP}=\beta_{kink}\times B\times e^{-\frac{\Delta G_{crit}}{kT}\times f\left(R^{\prime },m\right)},\;\Delta G_{crit}=\frac{16\pi \times \gamma_{OCP}^{3}\times V_{m}^{2}}{3\times {\left(RTln\left(\sigma_{OCP}\right)\right)}^{2}}$$

The nucleation rate ($J_{OCP}$) is proportional to a kinetic factor (B) and nucleation barrier $\left(\beta_{kink}\right)$ and is exponentially affected by the activation energy of nucleation ($\Delta G_{crit}$), which is determined by the interfacial tension ($\gamma_{OCP}=4.3\;\mathrm{mJ}/m^{2}\;$ [60]) between OCP and the solution, the supersaturation (σ) of OCP from Equation (8) and the contact angle function, $f\left(R^{\prime },m\right)$, for a nucleus on a substrate. The OCP molecular volume is determined as $V_{m}=\frac{V_{\mathrm{cell}}N_{A}}{Z}$, where $V_{cell}=$ 1220 Å^3^ is the OCP unit cell volume, Z = 2 is the number of formula units and $N_{A}$ is the Avogadro number. The geometrical factor ($\frac{16\pi }{3}$) represents spherical nuclei [7]. For *Model I*, $\beta_{kink}=1$.

We can compare the semi-empirical system Equation (9) and classical nucleation theory by approximating the region of high supersaturation using a linear function on the graph of the dependence of the growth rate of OCP crystals (ln(J)) calculated from Equation (9) on the undersaturation value (σ) in a double logarithmic scale (Figure 3j). This region can also be clearly seen on the graph of the dependence of the calculated nucleation rate on the time of immersion (Figure 3k). The results of approximation correspond to the values calculated by Equation (11) (Figure 3l), which confirms the equality of these models.

The impact of the factor $f\left(R^{\prime },m\right)$, i.e., whether DCPD is an optimal substrate for OCP crystallization, is worth discussing. Foreign particles are known to significantly reduce the nucleation barrier due to two factors: epitaxial and geometrical. This is described by the multiplier $f\left(R^{\prime },m\right)$ [59], where R’ is the ratio of the critical radius of the nucleus to the radius of curvature of the foreign particle. In our case, the substrate is represented by plates with a size of 19 μm, which is ≈10^5^ times more than the critical radius of 1.4 nm (taking $\sigma_{OCP}=2.4$ at the initial stage). Thus, the term R’→0. The epitaxial factor *m* is equal to m=(γsf−γcs)γcf=−0.9 (the values of $\gamma_{cs}=-0.32\;\frac{mJ}{m^{2}}\;\mathrm{and}\;\gamma_{cf}=4.3\;\frac{mJ}{m^{2}}$ correspond to the interfacial tension between the substrate DCPD and fluid, the crystal OCP and substrate DCPD, and the crystal OCP and fluid, respectively [60]), which indicates a complete mismatch between the crystal lattices of DCPD and OCP, which in good agreement with crystallographic data. Thus, R’→0, m→−1 and f→1 [59]. This means that the energy gain from OCP crystallization on the DCPD substrate is negligible. 

A similar experiment on the solubility of DCPD tablets in the SBF solution was carried out in the bioreactor to reveal the effect of mass transfer on the occurring processes. The results obtained (XRD and IR spectra and potentiometric and gravimetric measurements) fully match those for the static system. It was expected [53] that the dissolution rate of DCPD would be limited by the diffusion component. That is, the near-surface concentrations would rapidly reach saturation while the ions diffused slowly into the solution volume. However, in our case, the precipitation–dissolution processes most likely occurred in a thin surface layer; diffusion into the volume is of secondary importance. In addition, the mass transfer rate was much lower than the diffusion rate, which can also explain the coincidence of the results in static and dynamic inorganic systems. The experiment with the organic medium, however, was conducted in a bioreactor to facilitate the extrapolation of the experimental methodology to cellular studies, for which the presence of mass transfer is a critical condition.

### 3.3. Interaction of DCPD with Organic Solution

**Figure 4:**
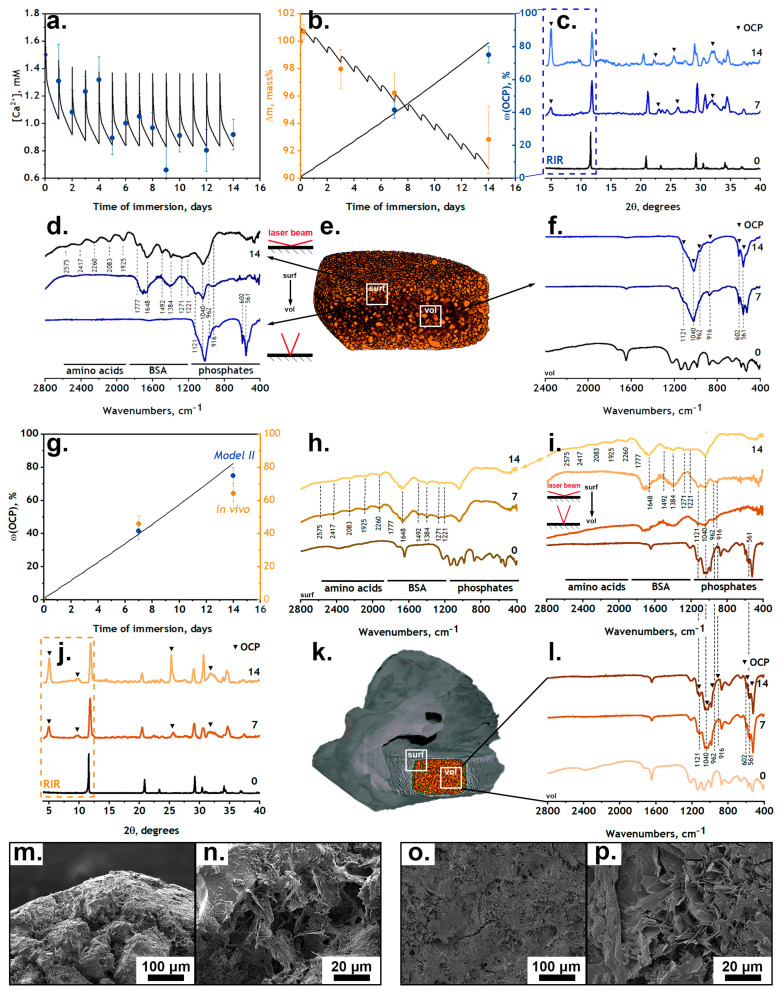
Interaction of DCPD with organic solutions: Scatters—experimental data; lines—calculations from *Model II*. (**a**) Concentration of free calcium ions in solution; (**b**) change in mass of samples and change in mass ratio of OCP in samples; (**c**) XRD results: 0-7-14 days of immersion; (**d**) FTIR spectra of material after 14 days of immersion, from surface to volume; (**e**) CT image of material after 14 days of immersion; (**f**) FTIR spectra of powder samples: 0-7-14 days of immersion; (**m**,**n**) SEM images of samples after 14 days of immersion. *In vivo* interaction between DCPD and organism: (**g**) change in mass ratio of OCP in samples compared with *Model II*; (**h**) FTIR spectra of material’s surface: 0-7-14 days of implantation; (**i**) FTIR spectra of material after 14 days of implantation, from surface to volume; (**j**) XRD results: 0-7-14 days of implantation; (**k**) CT image of material after 14 days of implantation; (**l**) FTIR spectra of powder samples: 0-7-14 days of implantation; (**o**,**p**) SEM images of samples after 14 days of implantation.

The incubation of samples in an organic medium (90% DMEM + 10% FBS) resulted in DCPD→OCP phase transformation accompanied by the chemical and morphological modification of the material surface (Figure 4c,d,f). Chemical modification included the adsorption of organic constituents (proteins and amino acids) by the surface, which was traced using the results of IR spectroscopy. At an angle of incidence of 10°, the spectrometer analyzed a 1–2 µm thick layer [61], and the spectrum clearly shows modes of albumin (in the region of 1220–1800 cm^−1^ [22]) and various amino acids (in the region of 1900–2500 cm^−1^ [62]). Increasing the angle of incidence to 80° increased the thickness of the analyzed layer, which led to a decrease in the mass fraction of organic components and, consequently, a decrease in the intensity of the modes at 1220–2500 cm^−1^ (Figure 4d). The spectra obtained from the sample volume are similar to those of the powdered samples: the presence of two calcium phosphate phases (DCPD and OCP) is evident, while the modes of organic components are not noticeable (Figure 4f).

The calcium concentration in the system decreasing due to the crystallization of OCP and the daily replacement of half the solution is well reflected by the calculated data (solid line, Figure 4a). The daily equilibrium calcium concentration reached a plateau in the region of 0.8 mM, which corresponds to the equilibrium value between the dissolution rate of DCPD and the crystallization rate of OCP. There is no pH measurement presented because the pH = 7.4 ± 0.1 was kept constant by the carbonate buffer system (the constant partial pressure of CO_2_ in the CO_2_ incubator). Rapid crystallization, and consequently the presence of extrema in Figure 4b in the first few hours, was observed during the experiment.

The addition of the organic compounds into the model solution led to the formation of large OCP aggregates with an average size of 15.1 ± 1.5 μm as opposed to 4.8 ± 0.6 μm in the SBF solution. The total surface area, according to BET nitrogen adsorption measurements, did not statistically change. It was shown that long thin OCP crystals were formed in SBF, exhibiting cytotoxicity [63]. At the same time, when conducting an experiment in a bioreactor in a DMEM+FBS medium, large flat crystals with adsorbed amino acids, integrins and proteins formed. Thus, the cellular responses to pure material, material soaked in SBF and material soaked in DMEM+FBS will be radically different from each other, and special attention should be paid to the incubation conditions.

Blood is composed of plasma and blood cells. Plasma can be divided into fibrinogen (clotting protein) and serum (inorganic salts, albumins, globulins, amino acids, hormones, etc.). The SBF solution used previously modeled the inorganic components of blood serum. A logical step to bring the model closer to the real conditions of the organism was to use a mixture of 10% blood serum (in our case, fetal (embryonic) bovine serum, FBS) and 90% culture medium, DMEM (Dulbecco Modified Eagle’s Medium), in the experiment. This solution composition would allow us to study the effect of organic components on the behavior of the material, and to include the cellular factor in the experiment without changing the solution composition.

Bovine serum albumin (BSA) is the dominant FBS protein (>50 mass.%) with a concentration of 30–60 mg/mL. A mixture of 10% FBS + 90% DMEM was used and the albumin concentration in it was determined colorimetrically as 3.2 ± 0.2 mg/mL (Bradford Protein Assay kit, Thermo Fisher Scientific, USA). BSA tends to form complexes with bivalent cations in solution. According to the literature [15], three sites for calcium binding are active in BSA under physiological conditions $\left(n_{1}=n_{2}=n_{3}=1\right)$ with the constants $k_{1}=361,$ $k_{2}=314$ and $k_{3}=291$. The total equation using Scratchard coordinates looks as follows: $\frac{\left[CaAlb\right]}{C\left(Alb\right)}=\overset{3}{\underset{1}{\sum }}\frac{n_{i}k_{i}\left[Ca\right]}{1+k_{i}\left[Ca\right]}$, where [CaAlb] is the equilibrium concentration of the albumin–calcium pair and C(Alb) is the total protein concentration. This summand should be added to Equation (2). The solution of the system of equations is the distribution of ionic forms in the DMEM-FBS mixture at the initial moment, presented in Figure 1b (taking into account 5% CO_2_ and pH = 7.4). In our case, less than 3 mol.% of all calcium ions were bonded to BSA, which is in good agreement with the data [64], taking into account the fact that we used a tenfold-diluted solution of FBS.

According to the literature data, the introduction of a protein component into the system does not directly affect the resorption kinetics of materials (in our case, DCPD), but inhibits the crystallization of the second phase (in our case, OCP) [64]. One way in which albumin may inhibit OCP crystallization is by adsorption onto the surface of the critical nuclei [23,59]. Thus, a multiplier $\beta_{kink}=e^{\left(\frac{\Delta G_{ad}}{RT}\right)}$ can be added to Equation (9) for the OCP deposition rate, responsible for increasing the energy barrier by increasing the value of the adsorption energy of serum albumin on the OCP surface. BSA adsorbs to the surface of OCP according to a Langmuir first-type isotherm with K = 1.52$\;\frac{ml}{mmol}\;\left[18,\;48\right],$ so $\Delta G_{ad}=-RTln\left(K\right),$ which corresponds to $\beta_{kink}=e^{\left(\frac{\Delta G_{ad}}{RT}\right)}\approx 0.656$. Similar values were obtained in [64]: the BSA addition to SBF led to a decrease in the OCP growth rate of up to 49.2%. This fact is well known in the literature: the introduction of a protein component allows for stabilizing extremely high supersaturation values for various CaPs in model solutions (spontaneous mineralization is prevented) in the absence of crystallization centers [23,24,25].

Using the initial conditions presented in Figure 1b, as well as constants from Section 3.2 and the energy barrier value $\beta_{kink}\approx 0.656$, we obtained

(12) $$\;\{\begin{array}{c} \frac{d\left[\mathrm{DCPD}\right]}{dt}=J_{\mathrm{DCPD}}=k_{1}s_{1}{\left(\sigma_{\mathrm{DCPD}}\right)}^{n1}\\ \frac{d\left[\mathrm{OCP}\right]}{dt}=J_{\mathrm{OCP}}=\beta_{kink}\times k_{2}s_{2}{\left(\sigma_{\mathrm{OCP}}\right)}^{n2} \end{array}$$

Equation (12) allows us to obtain a numerical model of the ongoing processes, in particular, the values of the free calcium concentration in the system and the amount of precipitated OCP over time (Figure 4a,b). It is worth noting that due to the increase in the energy barrier of nucleation, the reaction equilibrium shifts from nucleation to crystal growth, which leads to the formation of large OCP aggregates with an average size of 15.1 ± 1.5 μm as opposed to 4.8 ± 0.6 μm in the SBF solution. So, for $s_{1},$ Equation (10) was also used, but the total surface area, in this case, does not statistically change, i.e., $s_{2}=const$.

*Model II* is able to describe the main aspects of the bilateral material–medium interaction in a more complex dynamic organic system.

### 3.4. DCPD and Cell Line Interactions: Results of Cytotoxicity Analysis

Prior to *in vivo* studies, the cytotoxicity of this batch of materials was evaluated. The cytotoxicity of DCPD was studied in the mouse embryonic fibroblast cell line C3H/10T1/2 model, based on their staining with Hoechst 33,342 fluorescent dye (stains the nuclei of living and dead cells blue), propidium iodide (stains the nuclei of dead cells red) and calcein AM (stains the cytoplasm of living cells green). As a control group, cells were seeded on a culture plate without DCPD.

**Figure 5:**
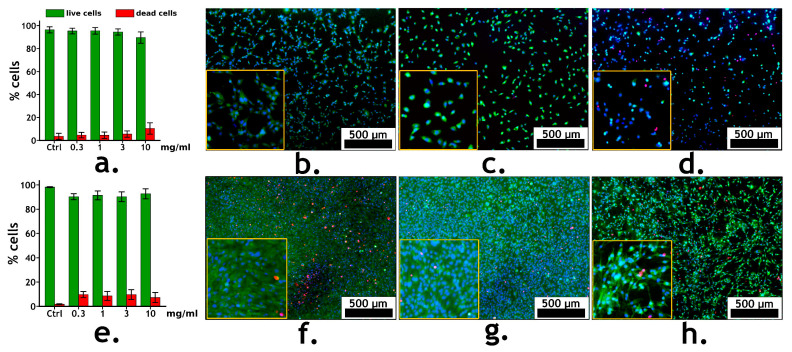
Cytotoxicity of different concentrations of DCPD after 24 (**a**) and 72 h (**e**) incubation with mouse embryonic fibroblast cell line C3H/10T1/2. (**b**–**d**) Fluorescence microimages of fibroblast cells C3H/10T1/2 in the control (**b**) and in the presence of 0.3 mg/mL DCPD (**c**) and 10 mg/mL DCPD (**d**) after 24 h of cultivation. (**f**–**h**) Fluorescence microimages of fibroblast cells C3H/10T1/2 in the control (**f**) and in the presence of 0.3 mg/mL DCPD (**g**) and 10 mg/mL DCPD (**h**) after 72 h of cultivation. Cell nuclei were stained with Hoechst 33,342 (blue), the cytoplasm of live cells was stained with calcein AM (green), and the nuclei of dead cells were stained with PI (red).

After culturing C3H/10T1/2 cells with DCPD at concentrations of 0.3, 1, 3 and 10 mg/mL for 24 h and 72 h, the number of dead cells did not differ statistically significantly from the control in the entire range of studied concentrations and for both observation periods (Figure 5a,e). At the same time, after 72 h of incubation with DCPD, the number of dead cells in the entire concentration range was slightly higher compared to after 24 h, but did not exceed 10% (Figure 5e). At the same time, taking into account the microscopic photographs, it is evident that when culturing with DCPD at the highest concentration of 10 mg/mL, the number of cells was slightly lower compared to the control conditions and did not reach a monolayer (Figure 5h). At the same time, a change in cell morphology occurred, consisting of the formation of elongated lamellipodia. For the data of other studied concentrations, the effect was not observed. This may indicate a stressful and cytostatic effect of high concentrations of DCPD on cells in the absence of a cytotoxic effect.

Thus, the obtained data indicate the absence of a cytotoxic effect of DCPD at all studied concentrations and, therefore, this material (and, in particular, this batch of material) is biocompatible. Consequently, DCPD, when implanted into the organisms of laboratory animals (Wistar stock rats), should not have any negative effect, and all the results obtained under *in vivo* conditions should maximally reflect the phase transformations of the material itself rather than the reaction of the foreign body in the recipient’s organism.

### 3.5. In Vivo Interaction Between DCPD and the Organism

According to the results of IR spectroscopy and XRD analysis, under the conditions of the subcutaneous implantation of the material in Wistar rats, a phase transformation of DCPD→OCP occurs (Figure 4h–j,l). It is noteworthy that chemically and morphologically the surface of the material after subcutaneous implantation is identical to that after the bioreactor experiment (identical IR spectra and crystal shape and size; Figure 4d vs. Figure 4i; Figure 4m,n vs. Figure 4o,p). The flat topography of the material after extraction observed in SEM images and the presence of crystals only in the recesses are related to the necessity to remove tissues fused to the surface of the material before SEM imaging, which slightly disturbed the real microstructure (Figure 4o). The unique surface layer tends to form in the first few days and does not change significantly when increasing the implantation time (Figure 4h), which indicates the stability of the surface layer and the further occurrence of reactions in the volume of the sample (Figure 4i). In addition, the rate of the phase transformation of DCPD→OCP is in good agreement with experimental data obtained in the bioreactor and using *Model II* (Figure 4g).

## 4. Discussion

The model presented in the current work is a generalization of many studies under conditions closer to physiological ones. In the process of developing the model, certain assumptions and simplifications were made, according to [3,4,7,8,9,10,52,53,54,55,56,57,58], as to what is necessary when describing complex systems such as a living organism. One of these assumptions was the usage of the Debye–Hückel equation to calculate ion activities. The main disadvantages of this equation include a limited descriptive ability with wide ranges of ionic strength values, an independence from temperature, and the lack of consideration of ion hydration. However, most living organisms are able to maintain the isotonicity of internal environments in narrow fixed ranges of ionic strength, which prevents osmotic shock and ensures temperature stability. One of the main advantages of this equation is its ease of use and low requirements for computing resources; therefore, at the cost of a small loss of accuracy, this equation describes systems similar to ours well [3,4,7,8,9,10,52,53,54,55,56,57,58]. In addition, this equation does not require knowledge of many specialized constants, as is the case in the specific theory of interactions (SIT) or in Pitzer’s theory [65].

The model we present is quite simple and does not take into account many of the factors listed above, and does not predict the size and shape of newly formed crystals, nor does it go into detail about the mechanisms of dissolution/crystallization. Therefore, it is necessary to clarify Equation (10) and determine the physical meaning of the *δ* parameter. More attention should be paid to diffusion, especially in conditions of fracture hematoma and fibronectin clot formation. A detailed study and numerical description of the influence of various organic components (amino acids, proteins, enzymes) on the behavior of CaP is necessary, since researchers have not yet numerically estimated the contribution of each of several options: adsorption on the surface of a critical nucleus, a decrease in the surface energy of a growing crystal, a local change in pH, an increase in viscosity in the near-surface layer and the chelation of calcium/phosphorus ions [7].

However, our goal was to create a system that was as simple as possible in terms of understanding and calculations, which would correlate with real conditions, which was achieved in this work. For the further development of the field of biomaterials science, scientists need to move from empirical comparisons of materials to quantitative assessments to numerical modeling.

The fact of changes in the morphology and chemical composition of the material surface can be considered another important conclusion. The primary cellular response to the material (such as the reaction of macrophages and monocytes or fibroblasts) should be tested *in vitro* on pure materials, but the direction of differentiation already needs to have been investigated on a specially treated surface. The direction of differentiation is affected by the morphology (nano- and microroughness) of the surface and the rigidity of the material, the presence of structural substitutions and the phase ratio in the case of polyphase samples [26,66,67]. Many scientific groups are trying to select a material that stimulates the osteogenic differentiation of stem cells; models have been developed that describe the mechanism of differentiation depending on the roughness and rigidity of the substrate surface [66], depending on the concentration of calcium in the surrounding fluid [67] and depending on the presence of external influences [34]. These models are of great value because they elaborate and explain complex processes in detail, but it is critically important to consider that the interaction of stem cells with the material under implantation conditions begins after a long period (>2 weeks). Since the body is a hostile environment for any material, the microstructure and phase composition of the implant can change significantly over some time after implantation, which was demonstrated in our work. Therefore, *in vitro* tests to study the osteoinductive properties of materials should be preceded by the incubation of the material under conditions close to those of the body, and SBF is not suitable for this.

## 5. Conclusions

The phase transformation of DCPD→OCP occurs in all observed *in vitro* and *in vivo* systems. It was shown that the usage of a bioreactor and a mixture of DMEM+FBS is crucial to the reproduction of surface modification. A surface layer forms on the first day and does not change during further incubation both in the organism and in the bioreactor, but it is significantly different in SBF. The *in vitro* experimental data proved to correlate perfectly with the results of the *in vivo* study. Although the biological processes occurring in the conditions of the organism have not yet been described mathematically, the results of *Model II* presented in this work fit well to *in vivo* tests.

The *in vitro* modeling of the primary alteration phase with a hematoma formation at the fracture site (the catabolic phase of regeneration) and the evaluation of its influence on the material–medium interactions are seen as a further development of this work. In our opinion, *Model II* is fundamental for understanding the influence of the phase and structural transformations of calcium phosphate materials in the example of DCPD on the initiation and character of the inflammatory stage of reparative osteogenesis and, consequently, on the specific course of the material and material-associated regeneration process in the recipient organism.

Thus, the obtained model test system can be a successful basis for a predictive high-content assessment of the efficacy and safety of biosynthetic materials *in vitro*. The proposed approach could be a step towards personalized medicine based on the patient’s biochemistry and medical history, using numerical modeling and/or experimental methods and selecting optimal drugs or implants. It can also reduce the number of experiments with animals and complies with the 3R principle (Replacement, Reduction, Refinement), and will be able to provide data on the response of a living organism without involving the organism itself. It fully complies with the new paradigm of the preclinical testing of new biosynthetic materials (animal-free, introduced by the FDA in 2023) through the use of human-on-a-chip technologies and organ and tissue biomimetics.

## Figures and Tables

**Table 1:** Equilibrium constants of ionic forms in solution at 37° C to calculate equilibrium concentrations [C_i_]; ionic composition of model solutions (theoretical concentrations) and characteristics of CaP tablets: average mass, specific surface area and average volume.

Equilibrium	lgK	Ref.		C, mM	Plasma	SBF	DMEM
HSO_4_^−^ = H^+^ + SO_4_^2−^	−1.94	[8]		**Na^+^**	142.0	142.6	153.0
H_3_PO_4_ = H^+^ + H_2_PO_4_^−^	−2.04	[42]		**K^+^**	5.0	5.0	5.4
H_2_PO_4_^−^ = H^+^ + HPO_4_^2−^	−6.7	[42]		**Mg^2+^**	1.5	1.5	0.5
HPO_4_^2−^ = H^+^ + PO_4_^3−^	−11.55	[42]		**Ca^2+^**	2.5	2.6	1.8
Ca^2+^ + HPO_4_^2−^ = CaHPO_4_ (aq)	1.3	[7]		**Cl^−^**	103.0	188.1	114.4
Ca^2+^ + H_2_PO_4_^−^ = CaH_2_PO_4_^+^	0.55	[7]		**HCO_3_^−^**	27.0	5.0	44.1
Ca^2+^ + SO_4_^2−^ = CaSO_4_ (aq)	4.43	[8]		**HPO_4_^2−^**	1.0	1.0	—
Ca^2+^ + HCO_3_^−^ = CaHCO_3_^+^	1.17	[7]		**H_2_PO_4_^−^**	—	—	0.9
Ca^2+^ + CO_3_^2−^ = CaCO_3_ (aq)	8.63	[7]		**SO_4_^2−^**	0.5	0.5	0.5
Mg^2+^ + HPO_4_^2−^ = MgHPO_4_ (aq)	1.8	[7]		**Buffer**	—	Tris	—
Mg^2+^ + H_2_PO_4_^−^ = MgH_2_PO_4_^+^	0.55	[7]		**I, M**	—	0.207	0.168
Mg^2+^ + SO_4_^2−^ = MgSO_4_ (aq)	2.37	[8]		**pH**	—	7.4	7.4
Mg^2+^ + HCO_3_^−^ = MgHCO_3_^+^	0.62	[7]		**S_DCPD_**	—	0.76	0.54
Mg^2+^ + CO_3_^2−^ = MgCO_3_ (aq)	1.87	[7]		**S_OCP_**	—	1.26	1.14
HCO_3_^–^ = H^+^ + CO_3_^2−^	−10.25	[7]					
Ca^2+^ + BSA_1_ = CaBSA_1_	2.56	[15]			m, g	s, m^2^/g	V, cm^3^
Ca^2+^ + BSA_2_ = CaBSA_2_	2.49	[15]		**DCPD**	0.6477 ± 0.0098	5.3 ± 0.3	76.9 ± 2.1
Ca^2+^ + BSA_3_ = CaBSA_3_	2.46	[15]					

**Table 2:** Observed and calculated XRD and NMR parameters.

	*№72-0713*	Full Profile	DFT	Site	δ iso (ppm)	
					Calc.	Exp.
**a**, Å	6.239	6.362 (3)	6.447	H_1_ (POH)	12.1	11.3
**b**, Å	15.180	15.1609 (24)	15.170	H_2_ (H_2_O)	7.0	6.4
**c**, Å	5.812	5.8083 (14)	5.903	H_3_ (H_2_O)	7.9	
**β**, °	119.232	118.545 (21)	119.33	H_4_ (H_2_O)	6.5	
**V**, Å^3^	493.93	492.13 (18)	498.47	H_5_ (H_2_O)	5.4	

## Data Availability

The original contributions presented in the study are included in the article, further inquiries can be directed to the corresponding authors.
